# Is There a Role for Topical Swallowed Steroids upon Emergency Room Admission for Suspected Food Bolus Obstruction in Eosinophilic Esophagitis?

**DOI:** 10.1007/s00455-021-10354-9

**Published:** 2021-08-16

**Authors:** Philipp Schreiner, Thomas Greuter, Aurora Tatu, Dagmar I. Keller, Alex Straumann, Luc Biedermann

**Affiliations:** 1 Department of Gastroenterology and Hepatology, University Hospital Zurich, Raemistrasse 100, 8091 Zurich, Switzerland; 2 Emergency Department, University Hospital Zurich, Zurich, Switzerland

**Keywords:** Esophageal food impaction, Budesonide orodispersible tablet, Deglutition, Deglutition disorders

## Abstract

Since most pharmacological treatments in case of esophageal food impaction (EFI) are unsuccessful, an endoscopy is usually required to resolve EFI. We present the first results of a budesonide orodispersible tablet (BOT) as a medical treatment option before endoscopy. We evaluated all patients with a suspected EFI to receive BOT before emergent endoscopy at a tertiary hospital between March 2019 and June 2020. A total of eight patients received BOT before endoscopy. Mean age was 50.1 years and 87.5% were male. In 38% (3/8) of patients the EFI resolved without endoscopic intervention. No adverse events occurred. After endoscopy, a diagnosis of EoE was established in 75%. This case series demonstrate the potential of BOT as medical rescue therapy in case of EFI.

## Introduction

Nowadays, eosinophilic esophagitis (EoE) represents the most common cause of esophageal food impaction (EFI) in patients presenting to the emergency room (ER) [1]. Despite conflicting evidence of pharmacological treatment options [2], several drugs have been suggested to avoid the invasive and costly endoscopy in EFI [3, 4].

Currently, the only approved medication for treatment of EoE is a budesonide orodispersible tablet formulation (BOT, Jorveza ®) showing high efficacy in induction [5] and in maintenance [6] treatment. Although the onset of action of locally administered glucocorticoids, including swallowed topical steroids (STCs) such as budesonide, in terms of genomic anti-inflammatory effects is typically within hours [7], recent data clearly demonstrate a non-genomic mode of action with a considerably more rapid onset of action within seconds to minutes [8]. It can be assumed that STC may exert rapidly anti-inflammatory effects and consequently diminish the local edematous reaction next to the bolus, especially in patients with an EoE without fibrostenotic phenotype. Additionally, according to Poiseuille’s law, resistance to flow in a tubular structure is inversely proportional to the 4th power of the radius, implying that already a minimal decrease in esophageal epithelial thickness is associated with considerable reduction in esophageal flow resistancy [9]. In view of these considerations, it is possible that an administration of BOT in the event of an EFI might promote resolution of bolus in patients with EoE.

The purpose of this report is to present the first experience using BOT as rescue treatment in clinically suspected, acute EFI.

## Methods

After the approval of BOT in Switzerland, physicians in the emergency department of the University Hospital of Zurich were instructed to directly administer one tablet of BOT (1 mg) to patients presenting with clinically suspected EFI in the absence of any previously known stricture in the ER and to inform the gastroenterologist on call. Whether BOT was administered was at the discretion of the gastroenterologist on duty. Patients aged 18 years or older, admitted to the emergency department between March 2019 and June 2020 with suspected EFI at the moment of presentation, were eligible to receive Jorveza® as a pre-endoscopy EFI management without delaying endoscopy.

Patients gave written informed consent in order to use the medical information.

## Results

**Fig. 1:**
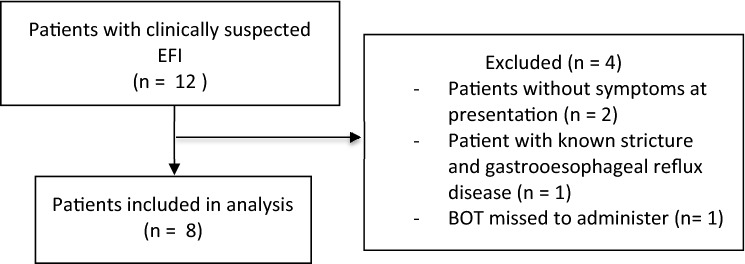
Flow chart

In 15 months, a total of twelve patients with clinically suspected EFI presenting to the ER were reported to the gastroenterologist on call of which eight patients received BOT containing 1 mg of Budesonide immediately after admission and thus were included in this analysis (Fig. 1). Mean age was 50.1 years (SD 11.4; 87.5% male). Mean time from onset of symptoms of impaction to admission was 7.9 h (SD 7.3 h), and from admission to complete resolution of symptoms or endoscopy 3.2 h (SD 2.2 h), respectively. None of the patients had an established diagnosis of EoE prior to endoscopy (Table 1).

**Table 1:** Patient characteristics

Patient	Gender	Age	Duration from Sx to admission (min)	Duration from admission to endoscopy/resolution of symptoms (min)	Type of food	Complete dysphagia	Intubation	Resolution of bolus before endoscopy	Diagnosis
1	m	42	360	480	Pizza	Yes	Yes	No	EoE with narrow-caliber esophagus
2	w	71	1320	120	Meat	No	No endoscopy	Yes	Loss to follow-up
3	m	40	345	75	Hazelnut	Yes	Yes	No	EoE
4	m	53	960	120	Meat	Yes	Yes	No	EoE-like disease with narrow-caliber esophagus
5	m	48	60	180	Shrimps	No	No	No	EoE
6	m	49	400	80	Meat	Yes	Yes	Yes	EoE
7	m	37	285	315	Meat	Yes	Yes	Yes	EoE
8	m	61	120	180	Meat	Yes	Yes	No	Reflux esophagitis with peptic stricture

Among eight patients who received BOT, in 38% the bolus could either not be observed in the esophagus at the time of endoscopy (2/8) or had complete resolution of symptoms so that scheduled emergent endoscopy was postponed (1/8). Endoscopic removal was necessary in five patients. In all patients with a documented stricture or narrow-caliber esophagus (3/8), persistent EFI was observed at endoscopy, requiring endoscopic removal. Among patients with a resolution of EFI before endoscopy, the type of food causing the bolus was in all cases meat. A clinico-histologic diagnosis of EoE was established in 75% (six out of eight patients).

## Discussion

Here we describe the first experience with BOT as medical rescue treatment in patients with clinically suspected EFI. In approximately 40% of patients with clinically suspected EFI the impaction has resolved after the administration of BOT.

However, due to the small sample size and the lack of a control group this brief report has several important limitations and the findings cannot provide any robust evidence on the efficacy of BOT in this indication. Nevertheless, the following considerations are arguments that STCs administered immediately upon presentation in the ER, before emergent endoscopy, might have the potential to promote resolution of EFI in EoE: firstly, the nearly 40% resolution rate in our case series is considerably higher compared to a study including 645 EFI, where a spontaneous resolution of impaction was found only in 16.7% [2], but similar to most used pharmacologic therapies in case of EFI [3]. Secondly, the time period between onset of EFI-indicating symptoms and ER admission was rather long; therefore, minimizing the probability of spontaneous resolution. Thirdly, in line with Poiseuille’s law only very subtle reductions of esophageal epithelial thickness (and thus esophageal lumen diameter) are associated with considerable reductions of flow resistance, potentially being sufficient to enable spontaneous bolus relief also in patients with a mixed fibrotic/inflammatory EoE phenotype.

Although efficacy of BOT does not appear to be superior to other pharmacologic therapies, the administration with an oral dissolution of a very small tablet (size 7.1 mm diameter) harbors almost no risk even in the emergency setting of an EFI. Of note, this intervention does not interfere with the established management of EFI; it has led neither to a delay nor to increased technical difficulties of the subsequent endoscopic procedure.

In conclusion, keeping in mind that emergency endoscopic procedures in EFI may not be promptly available in several institutions globally and harbor several substantial risks [3], and that the above mentioned medical intervention is almost riskless and has the potential to resolve food impactions, we believe that this approach is worth for further evaluation. A larger randomized placebo-controlled trial will provide definite clarification of BOTs efficacy in this indication.

## Abbreviations

BOT: Budesonide orodispersible tablet formulation
EFI: Esophageal food impaction
EoE: Eosinophilic esophagitis
ER: Emergency room
SD: Standard deviation
STC: Swallowed topical corticosteroids
